# Effects of additives in rehydrated corn silage with industrial tomato waste on intake, digestibility, ruminal parameters, and performance of feedlot-finished lambs

**DOI:** 10.1016/j.vas.2026.100591

**Published:** 2026-01-30

**Authors:** Patrick Ferreira Cardoso, João Paulo Sampaio Rigueira, Vicente Ribeiro Rocha Júnior, Jade Passos de Almeida, Sérgio da Conceição Assunção, Fredson Vieira e Silva, Tiago Alves Corrêa Carvalho da Silva, Thais Oliva Neres, Jordânia Pereira da Silva, Ronnie Antunes de Assis, Flávio Pinto Monção

**Affiliations:** a State University of Montes Claros, Department of Animal Science and Technology, Avenue Reinaldo Viana, 2630, Janaúba, Minas Gerais, Brazil; b University of New England, School of Environmental and Rural Science, Elm Avenue, NSW, 2351 Armidale, Australia

**Keywords:** Alternative feeding, Grain-rich diets, Industrial waste, Ovis aries

## Abstract

There are doubts as to whether or not rehydrated corn silage with industrial tomato waste (ITW) requires additives. Thus, study aimed to evaluate the effects of different additives in rehydrated corn silage with ITW on feed intake, nutrient digestibility, ruminal parameters, nitrogen balance, ingestive behavior, and performance of feedlot-finished lambs. Twenty-four intact Dorper × Santa Inês crossbred male lambs, averaging 20.88 ± 1.84 kg of initial body weight and 100 days of age, were used. Four experimental diets were tested: (1) rehydrated corn silage with ITW and without additives; (2) rehydrated corn silage with ITW and a bacterial-enzymatic inoculant; (3) rehydrated corn silage with ITW plus 2% powdered sugarcane molasses; and (4) rehydrated corn silage with ITW including both a bacterial-enzymatic inoculant and 2% powdered sugarcane molasses, on a natural matter basis. The roughage-to-concentrate ratio was 50:50, based on dry matter (DM). The experiment followed a completely randomized design with four treatments and six replicates. No differences were observed among diets in dry matter intake (*p*= 0.33), crude protein intake (*p*= 0.83), non-fibrous carbohydrate intake (*p*= 0.16), or total digestible nutrient intake (*p*= 0.92). Final body weight, average daily gain, hot carcass weight, hot carcass yield, and feed efficiency were 32.02 kg, 0.232 kg day⁻¹, 14.21 kg, 44.42%, and 0.23 kg of DM kg⁻¹, respectively. These findings indicate that adding bacterial-enzymatic inoculant or powdered sugarcane molasses to rehydrated corn silage with ITW is unnecessary in feedlot diets for finishing lambs.

## Highlights

• There was no difference in the performance of the lambs.
• Rehydrated corn with ITW does not require microbial additives.
• Industrial Tomato waste is a sustainable alternative nutrients source for ruminant production.

## 1. Introduction

In Brazil, ruminant production relies primarily on pastures as the main nutrient source throughout the year. This strategy lowers production costs compared with intensive systems. However, under tropical conditions, maintaining forage quality over extended periods is a major challenge, which complicates finishing animals on pasture.

Intensive finishing systems are therefore necessary, as they provide diets with higher energy density, largely derived from corn. Corn grown in Brazil is predominantly flint-type, which has low starch digestibility in the rumen and gastrointestinal tract. According to Jacovaci et al. (2021), Rosera et al. (2023), and Durães et al. (2024), starch in Brazilian corn is encapsulated in a protein matrix (prolamins), limiting hydration and enzymatic action, and resulting in high starch losses in feces. Fine grinding (1–2 mm), combined with rehydration and ensiling, has been proposed as a strategy to reduce starch losses. During ensiling, bacterial proteolysis of the protein matrix makes starch more available for ruminal degradation and digestion.

Although water is commonly used to rehydrate corn, alternative products such as ITW can serve the same purpose. ITW contains nitrogen compounds, non-forage fiber, bioactive compounds (carotenoids, vitamins E and C), and minerals, and lacks antinutritional factors, making it a potential feed ingredient (Silva et al., 2025a; Antunes et al., 2025, 2025b). Chemically, ITW contains 250 g/kg dry matter (DM), 190 g/kg DM crude protein, DM digestibility above 60 %, and a pH of 3. Improper disposal of ITW pollutes rivers, lakes, and dams (Das et al., 2018). However, its high moisture content (above 70 %), often cited by farmers as a limitation for storage and use, can be exploited as a means to rehydrate ground corn (Silva et al., 2025). This approach not only preserves the material for longer periods but may also enhance nutrient digestibility in Brazilian flint corn (Jacovaci et al., 2021).

Despite this potential, uncertainties remain. Both ITW and corn have low concentrations of water-soluble carbohydrates, and the epiphytic flora in these substrates raises concerns about fermentation quality (Diogénes et al., 2023; Silva et al., 2025). In this regard, there are doubts as to whether or not rehydrated corn silage with ITW requires additives. According to Antunes et al. (2025), rehydrated corn silage with ITW requires an enzymatic bacterial inoculant for better silage preservation. However, there are doubts as to whether the benefits observed in silages translate into better animal performance. The use of bacterial additives containing selected homofermentative and heterofermentative microorganisms, together with a source of water-soluble carbohydrates such as powdered sugarcane molasses, may reduce DM losses in silage and improve nutrient availability.

Given these considerations, we hypothesized that rehydrating corn grain with ITW combined with different additives could improve silage efficiency and animal performance, while enhancing nitrogen balance and dietary energy availability through better starch utilization. Therefore, our study was to evaluate the effects of different additives in rehydrated corn silage with ITW on feed intake, nutrient digestibility, ruminal parameters, nitrogen balance, ingestive behavior, and performance of feedlot-finished lambs in the Brazilian semiarid region.

## 2 Materials and methods

The experiment was conducted at the Experimental Shed for Animal Digestibility Tests, State University of Montes Claros, Janaúba, Minas Gerais, Brazil, located at 15°43′72.47″ S, 43°19′18″ W, and 516 m altitude.

### 2.1 Treatments and experimental design

Twenty-four uncastrated Dorper × Santa Inês crossbred male lambs, averaging 20.88 ± 1.84 kg initial body weight (median=20.50 kg, first quartile = 18.48 kg, and third quartile = 22.12 kg) and 100 days of age, were used. Four experimental diets were evaluated, differing only in the additives included in corn silage (ground corn grain, 1–2 mm, rehydrated with industrial tomato waste [ITW]). The treatments were: (1) Corn grain rehydrated with ITW and without additives; (2) Corn grain rehydrated with ITW plus a bacterial-enzymatic inoculant; (3) Corn grain rehydrated with ITW plus 2 % powdered sugarcane molasses; and (4) Corn grain rehydrated with ITW plus a bacterial-enzymatic inoculant and 2 % powdered sugarcane molasses, on a natural matter basis. The experiment followed a completely randomized design with four treatments and six replicates.

### 2.2 Silage management

Corn grain used for silage production was purchased locally and ground through a 1–2 mm sieve. Industrial tomato waste (ITW) was supplied by Bestpulp® (http://www.bestpulp.com.br/), located in Janaúba, Minas Gerais, Brazil (15°49′49″ S, 43°16′18″ W). Silage with 35 % moisture was produced (Antunes et al., 2025; Silva et al., 2025). For every 100 kg of ground corn, 46.80 kg of ITW was added. The density of the silos averaged 962.5 kg m^-3^. The mass was ensiled in silos made from 200-liter polyvinyl chloride drums. The silos remained sealed for 243 days.

Additives were included in treatments to improve silage fermentation. One gram of the lyophilized enzymatic-bacterial inoculant SILOTRATO™ was sprayed per ton of ground corn (natural matter), following the manufacturer’s recommendations. The manufacturer guaranteed the product’s compliance with quality standards. All treatments received 2 mL kg⁻¹ natural matter of dechlorinated water. The inoculant was evaluated for enzymatic activity and bacterial composition, independent of the manufacturer’s information. Powdered sugarcane molasses was added as a source of water-soluble carbohydrates. The inclusion of powdered sugarcane molasses was based on 2 % of the natural matter of the silage produced. Powdered sugarcane molasses was mixed in the ITW, and homogenized, then mixed with ground corn.

### 2.3 Animal and diet management

Animals were housed in individual pens (1.5 m²) equipped with feeders, drinkers, and slatted floors. The experimental period lasted 60 days, and was preceded by a 15-day adaptation period to diets, handling, and facilities. At the beginning of adaptation, lambs received an albendazole-based anthelmintic and a clostridial vaccine.

During feedlot, the diet was formulated both with a 50:50 roughage-to-concentrate ratio. Diets were designed to meet crude protein and metabolizable energy requirements for an average daily gain of 200 g during confinement, according to NRC (2007). Forage sorghum silage (BRS 658), harvested 120 days after planting, was used as the sole roughage source in all treatments. Rehydrated corn silages with ITW, soybean meal–based concentrate, and mineral mixture were offered twice daily (07:00 and 16:00 h) as a total mixed ration (TMR). Water was available *ad libitum*. Daily feed adjustments were based on orts weighing, maintaining 100 g kg⁻¹ refusals of the diet offered.

### 2.4 Chemical composition of ingredients

During experimental period, morning samples of feed offered, orts, and feces were collected and stored at –20 °C. Samples were later thawed, oven-dried at 55 °C for 72 h with forced air ventilation, and ground in a Wiley mill (MA340, Marconi, Piracicaba, Brazil) through 2- and 1-mm sieves.

Chemical and bromatological composition of feed, orts, and feces was analyzed at the Laboratory of Food Analysis and Animal Nutrition, Department of Agricultural Sciences, UNIMONTES. Analyses included dry matter (method 934.01, AOAC, 2012), total nitrogen by the Kjeldahl method (method 981.10, AOAC, 2012), crude protein (CP) content was calculated by multiplying total nitrogen content by 6.25. Additionally, ether extract analysis (method 945.16, AOAC, 2012), organic matter and mineral matter (method 930.05, AOAC, 2012). The neutral detergent fiber (NDF), and acid detergent fiber (ADF), with appropriate corrections for ash, protein, and lignin following the recommendations of Van Soest et al. (1991). For analyses of NDF and ADF heat-stable amylase were used. Total carbohydrates and non-fibrous carbohydrates were estimated according to Detmann et al. (2021). Total digestible nutrients (TDN) were calculated according to NRC (2001). Table 1 show the chemical composition of silage. Table 2, Table 3 show the ingredients and chemical composition of the experimental diets.

**Table 1:** Chemical composition of ingredients and silage.

Item (g kg^-1^ DM)^1^	Soybean Meal	Silage				
		BRS 658	Control	Inoculant	Molasses	Molasses with Inoculant
pH	-	4.01	4.16	4.13	4.20	4.15
Ammoniacal nitrogen, % TN	-	5.79	3.76	3.17	3.27	3.37
Lactic acid	-	69.7	20.9	22.1	24.5	22.5
Acetic acid	-	17.0	3.80	8.86	5.96	6.82
Propionic acid		4.60	0.87	5.26	1.66	1.73
Butyric acid	-	0.00	2.52	1.64	1.15	1.73
Ethanol	-	3.10	0.80	1.00	0.91	1.00
Dry matter	907	280	635	619	606	638
Ash	73.0	54.1	37.0	48.4	50.5	48.7
Organic matter	927	946	963	952	949	951
Crude protein	460	43.2	106	102	82.3	105
Ether Extract	21.5	17.8	136	65.2	83.3	96.1
Neutral detergent fiber	136	698	215	156	247	343
Neutral detergent fiber corrected for ash and protein	131	593	204	149	238	326
Acid detergent fiber	60.0	427	55.3	58.5	66.6	72.3
Total Carbohydrates	445	885	721	784	784	750
Non-fibrous carbohydrates	309	186	506	628	537	407
Total digestible nutrients	812	503	823	817	825	801

1 BRS 658 – BRS 658 forage sorghum silage; Control - Corn grain rehydrated with ITW and without additives; Inoculant - Corn grain rehydrated with ITW plus bacterial-enzymatic inoculant; Molasses - Corn grain rehydrated with ITW plus 2 % powdered sugarcane molasses; Molasses + Inoculant - Corn grain rehydrated with ITW plus bacterial-enzymatic inoculant and 2 % powdered sugarcane molasses, natural matter basis. For details on the fermentative parameters of silages, see the publication by Antunes et al. (2025).

**Table 2:** Ingredient proportions and chemical composition of the experimental diets.

Item	Experimental diet^1^			
	Control	Inoculant	Molasses	Molasses with Inoculant
Proportion of ingredients in the diets (g kg^-1^ dry matter)				
Sorghum Silage BRS 658	500	500	500	500
Rehydrated corn grain with ITW	250	250	250	250
Soybean meal	200	200	200	200
Mineral mix^2^	50.0	50.0	50.0	50.0
*Chemical composition (*g kg^-1^*dry matter)*				
Dry matter	530	526	523	531
Ash	101	104	104	104
Crude protein	140	139	134	140
Ether Extract	47.2	29.5	34.0	37.2
Total Carbohydrates	712	727	727	719
Non-fibrous carbohydrates	282	312	289	257
Neutral detergent fiber	430	415	438	462
Neutral detergent fiber corrected for ash and protein	387	374	394	416
Total digestible nutrients	620	618	620	614
Metabolizable energy, Mcal kg^-1^ DM	2.24	2.23	2.24	2.22

1 Control - Corn grain rehydrated with ITW and without additives; Inoculant - Corn grain rehydrated with ITW plus bacterial-enzymatic inoculant; Molasses - Corn grain rehydrated with ITW plus 2 % powdered sugarcane molasses; Molasses + Inoculant - Corn grain rehydrated with ITW plus bacterial-enzymatic inoculant and 2 % powdered sugarcane molasses, natural matter basis. ITW – industrial tomato waste.

2 Mineral mixture composition (per kg of product): calcium (135 g min), phosphorus (65 g min), sodium (107 g min), magnesium (6 g), sulfur (12 g), copper (100 mg), cobalt (175 mg), iron (1000 mg), iodine (175 mg), manganese (1440 mg), selenium (34 mg), zinc (6000 mg), and fluorine (650 mg).

**Table 3:** Chemical composition of ingredients used in silage production.

Item ( % DM)	Ground corn	Industrial tomato waste	Cane molasses
pH	6.80	3.00	6.90
Dry matter, % of natural matter	87.9	24.5	94.6
Ashes	1.48	4.46	4.85
Crude protein	9.28	19.5	3.52
Ether extract	3.53	9.07	0.85
Neutral detergent fiber	130	563	1.26
Neutral detergent fiber corrected for ash and protein	125	489	1.26
Acid detergent fiber	4.50	37.6	0.05
Indigestible dry matter	8.72	42.5	6.48
Indigestible neutral detergent fiber	3.68	14.2	1.84
Indigestible acid detergent fiber	1.66	8.17	0.59

DM – dry matter.

### 2.5 Nutrient intake and digestibility

Diet intake was monitored throughout the experimental period. Feed supply was adjusted daily to allow 10 % refusals (DM basis). Feeders were cleaned, and orts were weighed daily before morning feeding. Samples of feed offered and orts were collected every ten days and frozen at –20 °C for later DM determination. Daily dry matter intake (DMI) per animal was calculated as feed offered minus orts (DM basis).

During the 12 days of the experimental period, total feces and urine were collected. Samples of feed, orts, and feces were ground in a knife mill with a 2-mm sieve and incubated in the rumen of two adult crossbred steers (480 ± 30 kg body weight; 8 years old; cannulated) for 288 h, following Detmann et al. (2021), to estimate indigestible neutral detergent fiber (iNDF) as an internal marker. The digestibility coefficient of all nutrients was calculated by the following equation: [amount ingested - amount excreted in feces] / amount ingested. Based on the digestibility coefficients, the value of total digestible nutrients was calculated.

### 2.6 Nitrogen balance and ruminal parameters

Total urine was collected for 12 consecutive days during the experimental period. Nitrogen content was determined by the Kjeldahl method (method 981.10, AOAC, 2012). Nitrogen balance (g day⁻¹) was calculated as:

N retained (g) = {N ingested (g) - N fecal (g) - N urine (g)}, where: nitrogen balance = nitrogen retained in the animal's body; N ingested = nitrogen ingested by the animal; N fecal = nitrogen excreted in feces and N urine = nitrogen excreted in urine.

During the three days of the experimental period, ruminal pH, and ammonia nitrogen (N–NH₃) were measured. Ruminal fluid (100 mL) was collected from each animal (eleven o'clock in the morning; four hours after eating) by suction using an esophageal tube coupled to a vacuum pump. Samples were filtered through cotton cloth, homogenized, and immediately analyzed for pH with a benchtop pH meter (Microprocessor pH Meter R-TEC-7-MP). Ammonia nitrogen (N–NH₃) was determined according to Detmann et al. (2021) (INCT-CA N-007/2).

Volatile fatty acids (VFAs) were analyzed by high-performance liquid chromatography (Hewlett Packard 5890 Series II GC; CarboPack packed column, 3 m) equipped with an ultraviolet detector (Hewlett Packard 3396 Series II Integrator) and injector (Hewlett Packard 6890 Series), following Mathew et al. (1997).

### 2.7 Determination of ingestive behavior

Ingestive behavior was evaluated following the method of Mezzalira et al. (2011). Visual observations for each pen (*n*= 1) were recorded every 5 min over a 24-h period on days 19 and 20 of each experimental phase. Groups of trained observers were assigned to each 6-h interval, with each observer monitoring six pens (6 animals). Observations were performed sequentially and always in the same order. Feeding, rumination, and idling times (min day⁻¹) were calculated by multiplying the number of observations by 5.

### 2.8 Productive performance and slaughter

At the beginning and end of experimental period, after a 16-h fast, animals were weighed on an electronic scale (Valfran, Votuporanga, São Paulo, Brazil). Average daily gain (ADG) was calculated as the difference between final and initial body weights divided by days in confinement. Feed efficiency was calculated as ADG (kg day⁻¹) divided by DMI (kg day⁻¹).

At the end of the feedlot period, animals underwent a 16 h fasting period (solids only) and were then transported 20 km to a commercial abattoir, where slaughter followed national technical regulations (MAPA, 2021). Stunning was performed via electrical narcosis, followed by exsanguination and viscera removal. After slaughter, half-carcasses were weighed to obtain hot carcass weight, which was used to calculate hot carcass yield.

### 2.9 Multivariate analysis

A principal component analysis (PCA) was conducted to explore the relationships among the studied traits and independent variables. Thirty-seven characteristics were included in the analysis. Data were standardized to a mean of zero and variance of one, and a correlation matrix was used in place of a covariance matrix.

### 2.10 Statistical analysis

Data were analyzed by analysis of variance (ANOVA) using a model that included fixed effects of diets (silages). The UNIVARIATE procedure was applied to identify outliers, influential values, and to test residual normality. The statistical model was

Yij=μ+ti+Pj+eijk,

Where:

Yij = observed value for variable *i* in relation to treatment in the k^th^ repetition; *m* = mean of all experimental units for the variable under study; ti = effect of the experimental diets *i* on the value of observation Yijk;

Pj = effect of the initial body weight as a covariate; eijk = error associated with the independent observation Yijk, which, by hypothesis, has a normal distribution with mean equals to zero and variance of δ^2^.

When the F test indicated significance, means were separated using the Tukey test at α=0.05. Exploratory analyses, including principal component analysis (PCA) and cluster analysis, were performed using PAST® 4.03 software (Hammer et al., 2001). To calculate the power of the statistical test of each variable, the method of Cohen (1988) was used. In general, the results of the power of the statistical test were near than 0.7 (median = 0.67).

## 3 Results

### 3.1 Nutrient intake and digestibility

No differences were observed among diets for dry matter intake (*p*= 0.33), crude protein (*p*= 0.83), non-fibrous carbohydrates (*p*= 0.16), or total digestible nutrients (*p*= 0.92) in feedlot lambs. Mean values for these variables, in the same order, were 1000, 160, 310, and 670 g day⁻¹, respectively (Table 4).

**Table 4:** Nutrient intake and digestibility of feedlot lambs fed diets with corn grain rehydrated with industrial tomato waste plus different additives.

Item	Experimental Diet^1^				SEM	P-value
	Control	Inoculant	Molasses	Molasses with Inoculant		
	*Intake, g day^-1^*					
Dry matter	960	970	1040	1050	40.0	0.33
Crude protein	150	160	160	170	10.0	0.83
Ether extract	50a	30c	37b	40a	10.0	0.01
Neutral detergent fiber	380b	370b	420a	450a	20.0	0.01
Non-fibrous carbohydrates	300	310	350	310	10.0	0.16
Total digestible nutrients	660	690	680	650	40.0	0.92
	*Digestibility, g kg^-1^*					
Dry matter	644 a	699 a	605 b	585 b	2.68	0.01
Crude protein	662 a	711 a	606 b	542 b	3.43	<0.01
Ether extract	743 a	646 b	628 b	671 b	2.86	0.03
Neutral detergent fiber	436	477	320	372	4.9	0.12
Non-fibrous carbohydrates	954	976	988	999	1.45	0.09
Total digestible nutrients	684 a	702 a	627 b	618 b	2.36	0.03

Means followed by the same letter in a row do not differ from each other by the Tukey test (*P* < 0.05). SEM – Standard error of the mean; P- Probability.

1 Control- Corn grain rehydrated with ITW and without additives; Inoculant - Corn grain rehydrated with ITW plus bacterial-enzymatic inoculant; Molasses - Corn grain rehydrated with ITW plus 2 % powdered sugarcane molasses; Molasses with Inoculant - Corn grain rehydrated with ITW plus bacterial-enzymatic inoculant and 2 % powdered sugarcane molasses, natural matter basis. ITW – industrial tomato waste.

The inclusion of additives in rehydrated corn silage with ITW influenced ether extract and neutral detergent fiber intake. Lambs fed the control diet and the diet containing molasses with bacterial–enzymatic inoculant showed greater ether extract intake. Neutral detergent fiber intake was 14.22 % higher in lambs fed silages with molasses or with molasses plus bacterial–enzymatic inoculant compared with the other treatments.

Dietary treatments also affected nutrient digestibility. Dry matter (*p* = 0.01) and crude protein (*p* < 0.01) digestibility in lambs fed the control diet and the inoculant diet were 11.4 % and 16.4 % higher, respectively, than in the other treatments, with average values of 595 g kg^-1^ and 574 g kg^-1^. In contrast, neutral detergent fiber (*p* = 0.12) and carbohydrate (*p* = 0.09) digestibility were not affected, averaging 401 g kg^-1^ and 979 g kg^-1^, respectively.

### 3.2 Nitrogen balance and ruminal parameters

No differences were observed among diets in nitrogen (N) intake, which averaged 25.5 g day⁻¹ (Table 5). Fecal N concentration was highest and urinary N concentration lowest (*p*> 0.05) in lambs fed the diet with molasses plus inoculant. Nitrogen balance, expressed in grams and percentage, did not differ among treatments, averaging 21.7 g day⁻¹ and 84.1 %, respectively.

**Table 5:** Nitrogen balance and ruminal parameters of feedlot lambs fed diets containing corn grain rehydrated with industrial tomato waste plus different additives.

Item	Experimental Diet^1^				SEM	P-value
	Control	Inoculant	Molasses	Molasses with Inoculant		
*Nitrogen Balance*						
N-ingested, g day^-1^	24.7	25.1	25.6	26.6	1.50	0.82
N-feces, g day^-1^	2.27 b	2.41 b	2.44 b	2.65 a	0.06	<0.01
N-urine, g day^-1^	1.34 a	1.70 a	1.54 a	0.88 b	0.18	0.01
*Nitrogen Balance*, g day^-1^	21.1	21.0	21.6	23.1	1.49	0.73
*Nitrogen Balance*, %	84.2	82.1	83.8	86.2	1.35	0.20
*Ruminal parameters*						
pH	6.95	6.87	7.10	6.98	0.08	0.27
Ruminal ammonia nitrogen, mg dL^-1^	13.6	14.5	13.4	15.4	1.76	0.84
Acetate, % VFAs	70.8	72.1	71.2	71.8	0.84	0.71
Propionate, % VFAs	18.1	17.0	17.8	16.6	0.65	0.32
Butyrate, % AGV	11.0	10.9	10.9	11.6	0.74	0.87

Means followed by the same letter in the row do not differ from each other by the Tukey test (*P* < 0.05). SEM – Standard error of the mean; P- Probability.

1 Control- Corn grain rehydrated with ITW and without additives; Inoculant - Corn grain rehydrated with ITW plus bacterial-enzymatic inoculant; Molasses - Corn grain rehydrated with ITW plus 2 % powdered sugarcane molasses; Molasses with Inoculant - Corn grain rehydrated with ITW plus bacterial-enzymatic inoculant and 2 % powdered sugarcane molasses, natural matter basis. ITW – industrial tomato waste; VFAs - volatile fatty acids.

Ruminal parameters were also unaffected by dietary treatments. Mean ruminal pH was 6.97, and ammoniacal nitrogen concentration averaged 14.2 mg dL⁻¹. Similarly, no dietary effects were observed for volatile fatty acids. The mean molar proportions of acetic, propionic, and butyric acids were 71.5 %, 17.4 %, and 11.1 % of total fatty acids, respectively.

### 3.3 Ingestive behavior

No effects of diet were observed on ingestive behavior variables in feedlot-finished lambs (Table 6). On average, animals spent 318 min feeding, 395 min ruminating, and 727 min idling. Rumination frequency averaged 11.90 chews min⁻¹. Diets influenced (*p*< 0.05) the number of rumination chews per bolus. Lambs fed the control diet and the molasses diet showed higher values (mean = 57.04 chews bolus⁻¹), 7.55 % greater than the average of the other treatments. Feed efficiency of dry matter and neutral detergent fiber was not affected by dietary treatments.

**Table 6:** Ingestive behavior of feedlot lambs fed diets with corn grain rehydrated with industrial tomato waste plus different additives.

Item	Experimental Diet^1^				SEM	P-value
	Control	Inoculant	Molasses	Molasses with Inoculant		
Eating, minutes	303	303	337	328	15.6	0.32
Rumination, minutes	402	350.4	420	410	24.6	0.18
Idleness, minutes	735	787	683	703	31.8	0.33
Total chewing, chews min⁻¹	11.7	10.9	12.7	12.3	0.50	0.09
Total chewing/bolo	57.5 a	52.0 b	56.6 a	53.4 b	1.53	0.05
*Intake efficiency (g* min*^-1^)*						
Dry matter	194	206	190	204	13.89	0.83
Neutral detergent fiber	76.9	78.1	75.7	87.8	5.65	0.42
*Rumination efficiency, (g* min*^-^*^1^*)*						
Dry matter	162	174	151	162	11.42	0.59
Neutral detergent fiber	64.1	65.9	60.7	70.3	4.71	0.53

1 Control- Corn grain rehydrated with ITW and without additives; Inoculant - Corn grain rehydrated with ITW plus bacterial-enzymatic inoculant; Molasses - Corn grain rehydrated with ITW plus 2 % powdered sugarcane molasses; Molasses with Inoculant - Corn grain rehydrated with ITW plus bacterial-enzymatic inoculant and 2 % powdered sugarcane molasses, natural matter basis. ITW – industrial tomato waste; Means followed by the same letter in a row do not differ from each other by the Tukey test (*P* < 0.05). SEM – Standard error of the mean; P- Probability.

### 3.4 Productive performance and slaughter

No dietary effects (*p*> 0.05) were observed on performance variables (Table 7). Final body weight, average daily gain, hot carcass weight, hot carcass yield, and feed efficiency averaged 32.0 kg, 0.232 kg day⁻¹, 14.2 kg, 44.4 %, and 0.23 kg DM kg⁻¹ ADG, respectively.

**Table 7:** Body performance of feedlot lambs fed diets with corn grain rehydrated with industrial tomato waste plus different additives.

Item	Experimental Diet^1^				SEM	P-value
	Control	Inoculant	Molasses	Molasses with Inoculant		
Initial Body Weight, kg	20.8	21.1	21.2	20.4	1.84	0.98
Final Body Weight. kg	31.3	33.2	32.7	30.9	2.02	0.81
Daily weight gain, kg	0.22	0.25	0.24	0.22	0.01	0.37
Hot carcass weight, kg	14.1	15.2	14.1	13.3	0.91	0.51
Hot carcass yield, %	45.1	46.0	43.2	43.3	1.12	0.23
Feed Efficiency, kg of DM kg^-1^ of ADG	0.23	0.26	0.23	0.21	0.02	0.49

1 Control- Corn grain rehydrated with ITW and without additives; Inoculant - Corn grain rehydrated with ITW and bacterial-enzymatic inoculant; Molasses - Corn grain rehydrated with ITW plus 2 % powdered sugarcane molasses; Molasses with Inoculant - Corn grain rehydrated with ITW plus bacterial-enzymatic inoculant and 2 % powdered sugarcane molasses, natural matter basis. ITW – industrial tomato waste; ADG - average daily weight gain; SEM – Standard error of the mean; P- Probability.

**Fig. 1:**
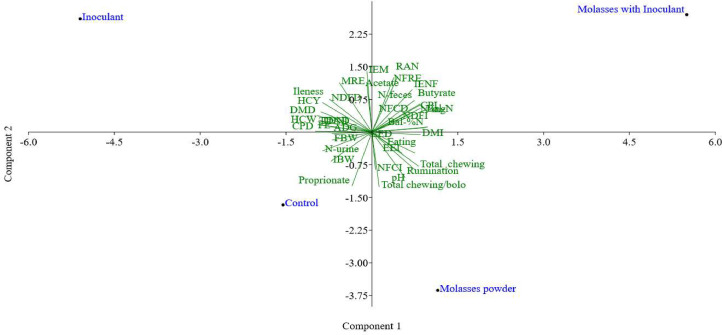
Schematic representation of the first (PC1) and second (PC2) principal components of dependent variables in feedlot lambs fed diets with corn grain rehydrated with industrial tomato waste plus different additives.

Based on the correlation matrix, principal components (PC) 1 and 2 explained 81.08 % of the total variation (Fig. 1). PC1 accounted for 54.04 % of the variation, with the highest correlations observed for crude protein digestibility (–0.2230) and non-fibrous carbohydrate digestibility (+0.2119). When PC1 and PC2 were considered together, higher coefficients were observed for ruminal ammonia nitrogen (+0.0997; +0.2821) and rumination efficiency of neutral detergent fiber (+0.0861; +0.2827). These traits, represented in PC 1 and PC2, were most associated with the molasses plus bacterial–enzymatic inoculant diet, which presented the highest coefficient (+ 5.5017).

**Fig. 2:**
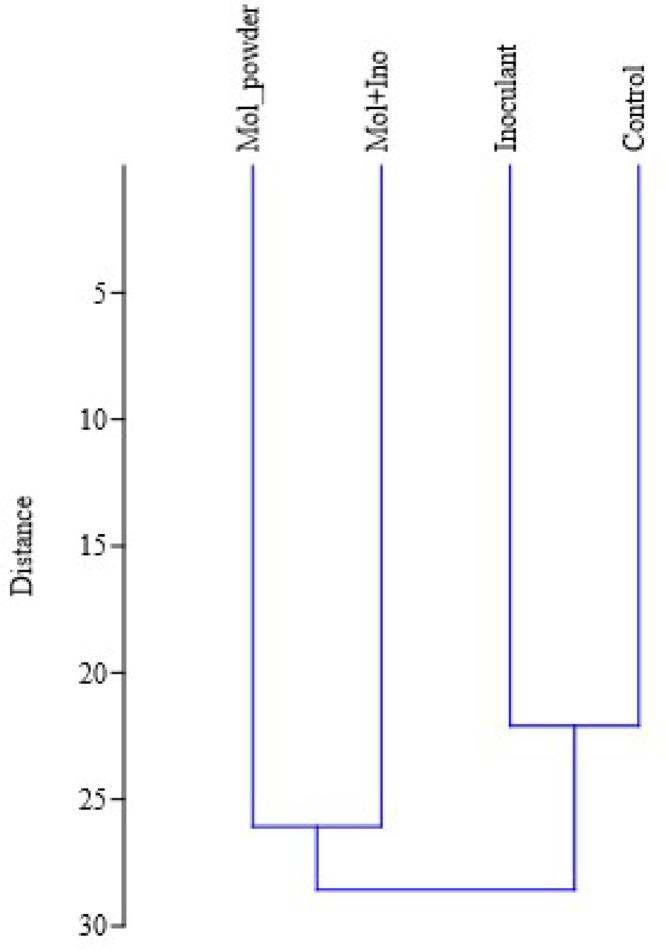
Cluster analysis of the general behavior of variables across experimental diets. Control - Corn grain rehydrated with ITW and without additives; Inoculant - Corn grain rehydrated with ITW and bacterial-enzymatic inoculant; Mol_powder - Corn grain rehydrated with ITW plus 2 % powdered sugarcane molasses; Mol+Ino - Corn grain rehydrated with ITW plus bacterial-enzymatic inoculant and 2 % powdered sugarcane molasses, natural matter basis. ITW – Industrial tomato waste.

Cluster analysis (Fig. 2) showed that lambs fed the control and inoculant diets exhibited similar patterns, with smaller Euclidean distances compared with diets containing molasses or molasses plus inoculant.

## 4 Discussion

According to Antunes et al. (2025) and Silva et al. (2025), ITW can be used to rehydrate ground corn for ensiling. According to Antunes et al. (2025), rehydrated corn silage with ITW associated with bacterial-enzymatic inoculant showed better fermentative and nutritional parameters. However, a knowledge gap remains as to whether the benefits observed in silage with the use of bacterial-enzymatic inoculants have effects on animal performance.

In this study, dry matter intake averaged 1000 g day⁻¹, consistent with NRC (2007) recommendations. The absence of dietary effects on dry matter and nutrient intake (CP, NDF, NFC, and TDN) can be attributed to the similar composition of the experimental diets, all of which contained equal proportions of corn rehydrated with ITW. This results can be confirmed by the similarity in the fermentative parameters of rehydrated corn silages with ITW associated with the additives. Powdered cane molasses can improve dry matter intake by animals. This occurs mainly by increasing palatability and aroma in the diet, making it more attractive for consumption. However, this effect was not observed in lambs in confinement.

Crude protein intake in this study (mean = 160 g day⁻¹) exceeded the NRC (2007) requirement of 101 g day⁻¹ for lambs averaging 20 kg body weight and gaining 200 g day⁻¹. Likewise, metabolizable energy intake (2.41 Mcal kg⁻¹ DM; 66.7 % TDN) surpassed the NRC (2007) estimate of 2.35 Mcal kg⁻¹ DM for this category. This nutrient supply, particularly of protein and energy, supports the performance observed, with several animals exceeding 200 g day⁻¹ average daily gain.

Greater ether extract intake was observed in lambs fed the control diet and the diet with molasses plus inoculant. This response may be explained by the higher concentration of crude fat in these two experimental diets (Table 2). Although the rehydrated corn silages presented the same proportion of ITW, this variation in ether extract content in the experimental diets and silages can be attributed to the residue. ITW is mainly composed of tomato husks and seeds. The seeds are rich in crude fat. During the processing of ITW for the production of rehydrated corn silages, unintentional selection of a higher proportion of seeds may have occurred in the control treatment and in the treatment with powdered cane molasses. This allowed for this variation in the chemical composition of the rehydrated corn silages with ITW associated with additives. The additives used, biologically, do not have the potential to increase the ether extract content due to their chemical composition. Despite the increased ether extract intake, total digestible nutrient intake did not differ among diets.

Dry matter and crude protein digestibility differed among diets; however, these variations did not affect nutrient intake or animal performance. This indicates that the differences in digestibility were not sufficient to alter these parameters. Typically, lower dry matter intake enhances digestibility because feed remains longer in the rumen, but this was not observed in the present study. According to Mertens (1994), diets with high digestibility and low rumen fill are consumed according to the animal’s energy demand. Thus, the lower dry matter intake in lambs fed the control and inoculant diets may have been related to the higher TDN content of these diets, but this was not confirmed.

Nitrogen balance in lambs is a key metric for evaluating diet efficiency, optimizing muscle growth, and increasing body weight gain. Nitrogen balance is influenced by the quality and quantity of crude protein, concentrates, and roughage in the diet. In this research, the nitrogen balance in the animals did not differ among diets. The diet containing silages with molasses or with molasses plus bacterial-enzymatic inoculant showed lower crude protein digestibility compared to the other treatments, however, this variation did not affect the nitrogen intake and neither the nitrogen balance by the animals. Animals fed a diet containing silages with molasses plus a bacterial-enzymatic inoculant showed greater fecal nitrogen loss. Fecal nitrogen is derived from undegraded protein in the rumen, demonstrating a small imbalance between rumen-degraded protein and digestible organic matter.

In general, there is no difference in dry matter intake and nitrogen balance among the animals suggesting that fermentation of corn during ensiling disrupted the protein matrix (prolamins) surrounding starch, thereby releasing carbon skeletons for microbial protein synthesis (Ferraretto et al., 2018; Cruz et al., 2021; Rosera et al., 2023; Durães et al., 2024). When carbon skeletons are not available, changes in nitrogen balance may occur in animals fed rehydrated corn silages (Soares et al., 2024). Although no differences were detected in nitrogen balance, the values obtained indicate constant nitrogen metabolism (NRC, 2007).

Urinary nitrogen reflects protein degraded in the rumen by bacterial metabolism, whereas fecal nitrogen corresponds to indigestible protein not degraded in the rumen. The influence of diets on urinary and fecal N excretion suggests that, four hours after feeding, excess protein degradation may have occurred with higher feed intake during this period. However, because nitrogen balance was similar across treatments, there was likely no imbalance between rumen-degraded protein and digestible organic matter. The absence of dietary effects on nitrogen balance and ruminal parameters may explain the similar dry matter intake observed among treatments.

No differences among diets were observed for ruminal pH and ammonia nitrogen (N–NH₃), which averaged 6.97 and 14.22 mg dL⁻¹, respectively. Although values did not vary across treatments, they provide insight into ruminal health. A ruminal pH below 5.6 is often used as a threshold for chronic acidosis (Cooper et al., 1997). This is relevant given that lambs consumed diets with 52 % concentrate, rich in starch. Starch, a glucose homopolymer, supplies carbon skeletons for microbial protein synthesis when nitrogen is not limiting. When starch is fermented to propionic acid, lactic acid may also be produced via the acrylate pathway; because lactate has a low pKa, it can decrease ruminal pH.

The stable pH values observed here may be explained by the amount of roughage (500 g kg⁻¹ DM) provided in all treatments. Dietary fiber stimulates chewing and saliva secretion, which buffer rumen pH (NRC, 2001). Additionally, the similar levels of non-fibrous carbohydrates among diets, and the efficiency of NDF in all silages to stimulate rumination and salivation (Lechartier & Peyraud, 2010), contributed to stable ruminal pH despite differences in additive use. Ruminal ammonia values, which did not differ among treatments.

The ruminal concentrations of N–NH₃ measured in this study fall within the range considered adequate (5–80 mg dL⁻¹) for microbial protein synthesis (Satter & Roffler, 1975; McDonald et al., 2011). Fluctuations in ruminal ammonia depend on the synchronization of energy and protein degradation rates (Piran Filho et al., 2023). A failure in this synchronization could increase ruminal ammonia concentration. Lechartier and Peyraud (2010) reported that diets with rapid ruminal degradability result in lower N–NH₃ concentrations than those with slower degradability, and that increasing dietary concentrate is associated with higher ruminal ammonia. As forage levels were maintained in this study, no such effect occurred. These results indicate adequate synchronization between energy and protein.

In general, the use of additives in rehydrated corn silage with ITW appears to be more effective for improving fermentation parameters, as reported by Antunes et al. (2025). However, their effects on energy metabolism were similar across diets and did not influence ingestive behavior or animal performance.

Multivariate analysis highlighted ammoniacal nitrogen concentration as one of the most influential variables. In the joint evaluation of PC1 and PC2, the molasses plus inoculant diet showed the highest N–NH₃ concentration. Ammonia nitrogen is fundamental in the rumen, as it is the main source of nitrogen used by ruminal microorganisms for the synthesis of high-quality microbial protein (Durães et al., 2024). The diet containing silages with molasses plus bacterial-enzymatic inoculant resulted in a higher ruminal concentration of ammonia nitrogen (mean of 15.4 mg dL^-1^) based on multivariate analysis. The ideal concentration of ammonia nitrogen in the ruminal fluid varies between 5 and 20 mg dL^-1^ to maximize microbial synthesis, indicating that there was no excess in the diet with molasses plus bacterial-enzymatic inoculant. This higher concentration indicates that the crude protein in the diet with molasses plus bacterial-enzymatic inoculant has a greater possibility of being hydrolyzed in the rumen and generating peptides and amino acids, which in turn can undergo deamination, releasing ruminal ammonia nitrogen (Van Soest, 1994). On the other hand, a lack of ruminal ammonia limits microbial growth and activity, impairing fiber digestion and protein synthesis, which reduces dry matter intake and animal performance. Excess ammonia is absorbed by the ruminal wall, transported to the liver, and converted into urea for excretion in the urine. At very high concentrations, which were not verified in this research, ammonia can be toxic and cause ruminal and metabolic alkalosis, leading to urea poisoning.

Cluster analysis indicated that control and inoculant diets were more similar to each other, with smaller Euclidean distances, compared with the molasses treatments. This suggests that these diets share closer relationships in nutrient dynamics and performance outcomes, making them more relevant in comparative evaluation. In the principal component analysis, dependent variables such as hot carcass weight, final weight, dry matter digestibility, crude protein digestibility, neutral detergent fiber digestibility, and feed efficiency were identified for animals fed a diet of rehydrated corn silage with ITW combined with an inoculant. Despite the lower coefficients for these dependent variables compared to ammonia nitrogen, they should be considered for decision-making. Based on cluster analysis, the decision regarding the best treatment lies in whether or not to use the bacterial-enzymatic inoculant for the production of rehydrated corn silage. According to Antunes et al. (2025), rehydrated corn silage with ITW combined with an inoculant showed better results regarding the fermentative and nutritional parameters of the silages. In practice, ensiling processes are fundamental to obtaining high-quality silage. The decision to use or not use bacterial-enzymatic inoculant in silage will depend on costs and objectives.

## 5 Conclusion

The use of bacterial-enzymatic inoculant or powdered sugarcane molasses in the ensilage of ground corn grain rehydrated with industrial tomato waste in the diet of lambs finished in feedlot does not alter dry matter intake, ruminal parameters, nitrogen balance, ingestive behavior and animal performance.

However, considering the importance of using bacterial-enzymatic inoculant to reduce dry matter losses in rehydrated corn silage, as well as the low acquisition cost, the authors recommend their inclusion.

## Ethics approval

The procedures used in this experiment were approved by the Ethics Committee on Animal Use (CEUA) under protocol no 011/2023.

## Data and model availability statement

The datasets generated and analyzed during the current study are not publicly available but are available from the corresponding author on reasonable request.

## Declaration of generative AI and AI-assisted technologies in the writing process

During the preparation of this work, the author(s) no used AI-assisted technologies for English editing purposes, including checking for grammatical errors and correcting misplaced punctuation.

## CRediT authorship contribution statement

**Patrick Ferreira Cardoso:** Writing – original draft, Methodology, Formal analysis, Conceptualization. **João Paulo Sampaio Rigueira:** Writing – review & editing, Writing – original draft, Validation, Methodology, Formal analysis, Data curation, Conceptualization. **Vicente Ribeiro Rocha Júnior:** Writing – review & editing, Writing – original draft, Validation, Supervision, Project administration, Methodology, Formal analysis, Conceptualization. **Jade Passos de Almeida:** Writing – review & editing, Writing – original draft. **Sérgio da Conceição Assunção:** Writing – review & editing, Writing – original draft, Methodology, Formal analysis. **Fredson Vieira e Silva:** Writing – review & editing, Writing – original draft. **Tiago Alves Corrêa Carvalho da Silva:** Writing – review & editing, Writing – original draft. **Thais Oliva Neres:** Writing – review & editing, Writing – original draft, Methodology, Formal analysis. **Jordânia Pereira da Silva:** Writing – original draft, Methodology, Formal analysis, Data curation. **Ronnie Antunes de Assis:** Writing – review & editing, Writing – original draft. **Flávio Pinto Monção:** Writing – review & editing, Writing – original draft, Visualization, Validation, Supervision, Project administration, Methodology, Investigation, Formal analysis, Data curation, Conceptualization.

## Declaration of competing interest

The authors wish to acknowledge that there are no known conflicts of interest related to this paper and there has been no financial assistance for this research that could have impressed its outcome.
